# Comparative maternal-fetal outcomes associated with different antihypertensive treatment strategies in preeclampsia: a retrospective cohort study

**DOI:** 10.3389/fphar.2026.1832562

**Published:** 2026-05-22

**Authors:** Lin Zhao, Rui Chen, Yahui Xu, Haiying Wu

**Affiliations:** Department of Obstetrics, Henan Provincial People’s Hospital, Zhengzhou, Henan, China

**Keywords:** antihypertensive therapy, labetalol, maternal-fetal outcomes, nifedipine, preeclampsia

## Abstract

**Background:**

Preeclampsia seriously threatens maternal and infant health. Antihypertensive therapy is key to improving perinatal outcomes, yet comparative maternal-fetal evidence for oral agents remains limited.

**Objective:**

To compare maternal–fetal outcomes of oral labetalol versus oral nifedipine in preeclampsia, informing individualized clinical treatment.

**Methods:**

This retrospective cohort study included consecutive preeclampsia patients admitted from January 2023 to December 2025, divided into Labetalol (n = 154) and nifedipine (n = 136) groups. Both received magnesium sulfate as needed, plus aspirin or low-molecular-weight heparin based on platelet count and coagulation function. After 1:1 propensity score matching (PSM), maternal and fetal outcomes were compared. Primary outcomes were maternal complications and neonatal outcomes. Secondary outcomes included uterine artery blood flow, hemodynamics, fetal growth restriction, and adverse drug reactions.

**Results:**

Following PSM, baseline characteristics were comparable between the two groups (P > 0.05). Both achieved similar improvements in blood pressure, uterine artery blood flow (S/D, PI, RI), and hemodynamic indicators (plasma and whole blood viscosity, hematocrit) (P > 0.05). The Labetalol Group, compared to the nifedipine Group, had significantly lower rates of postpartum hemorrhage (8.8% vs. 17.0%, P = 0.043) and preterm birth (24.6% vs. 37.3%, P = 0.041), and higher neonatal birth weight (2933.95 ± 803.23 g vs. 2541.35 ± 631.41 g, P < 0.001). Conversely, the nifedipine Group experienced higher incidences of headache (16.4% vs. 7.3%, P = 0.037) and facial flushing (13.6% vs. 4.6%, P = 0.019). Multivariate Logistic regression identified maternal age ≥35 years, pre-pregnancy overweight/obesity, preeclampsia onset at <34 weeks, primiparity, multiple pregnancy, severe preeclampsia, and pre-gestational diabetes as independent risk factors for adverse maternal-fetal outcomes (all P < 0.05).

**Conclusion:**

Both labetalol and nifedipine effectively control blood pressure and improve uteroplacental blood flow and most maternal-fetal outcomes in patients with preeclampsia. Adverse effects (headache and facial flushing) were more commonly observed with nifedipine, whereas labetalol was associated with a lower incidence of preterm birth and postpartum hemorrhage. These findings suggest potential advantages of labetalol in specific outcomes, but causal inferences are limited by the observational study design.

## 1 Introduction

A serious threat to maternal and infant health, preeclampsia is a pregnancy-specific disorder defined by new-onset hypertension and proteinuria or end-organ dysfunction after 20 weeks of gestation (Rosenberg and Seely, 2024). Global epidemiological data indicate that the incidence of preeclampsia ranges from 1.0% to 5.6%, with an incidence of approximately 2.4% in China and a total incidence of hypertensive disorders of pregnancy reaching 9.5% (Liu et al., 2021). Preeclampsia can lead to multiple organ dysfunction in the mother, including liver abnormalities, renal failure, coagulation disorders, and neurological complications. In severe cases, it can progress to eclampsia, HELLP syndrome, or even maternal death (Chiang et al., 2024; Preeclampsia, 2024; Countouris and Bello, 2025). Although termination of pregnancy is the only definitive cure for preeclampsia, active antihypertensive therapy, spasmolysis, and close monitoring are core components in improving perinatal outcomes before the optimal time for delivery is reached (Wu et al., 2023). Therefore, optimizing antihypertensive treatment strategies and selecting safe and effective antihypertensive medications are of significant clinical importance for delaying disease progression, prolonging gestation, and improving maternal and fetal prognosis.

Currently, both labetalol and nifedipine are recommended as first-line agents for antihypertensive therapy in preeclampsia by international and domestic guidelines (Scott et al., 2022). Extensive clinical evidence shows both drug classes lower blood pressure and delay progression in preeclamptic patients. However, existing comparative studies often focus primarily on the antihypertensive efficacy itself, with a lack of systematic evaluation of comprehensive maternal-fetal outcomes, including maternal hemodynamic changes, uterine artery perfusion status, and neonatal outcomes, under different medication strategies (S et al., 2022; Sanusi et al., 2024). The goal of preeclampsia management extends beyond merely controlling blood pressure; it aims to delay disease progression, reduce maternal complications, and improve fetal prognosis. Uterine artery hemodynamic parameters, key indicators of placental perfusion, are closely linked to adverse outcomes. Therefore, evaluating antihypertensive effects alone is no longer sufficient to comprehensively guide individualized clinical medication choices. In-depth investigation into the differential impacts of various antihypertensive regimens on comprehensive maternal-fetal outcomes—particularly their effects on uteroplacental blood perfusion, the risk of postpartum hemorrhage, the incidence of preterm birth, and neonatal prognosis—is of paramount importance for achieving precision treatment and improving overall pregnancy outcomes.

In light of this, the present study employed a retrospective cohort design, utilizing Propensity Score Matching (PSM) to control for confounding factors. It systematically compared the effects of two antihypertensive regimens—oral labetalol versus oral sustained-release nifedipine tablets—on maternal complications, uteroplacental hemodynamic parameters, and neonatal outcomes in patients with preeclampsia. Furthermore, it investigated independent predictors of adverse maternal-fetal outcomes. This study aims to deepen the understanding of the hemodynamic effects and maternal-fetal safety profiles of different antihypertensive agents, thereby providing direct comparative evidence to inform the individualized selection of oral antihypertensive therapy for patients with preeclampsia, ultimately optimizing perinatal management and improving maternal and fetal prognosis.

## 2 Methods

### 2.1 Participants

**FIGURE 1:**
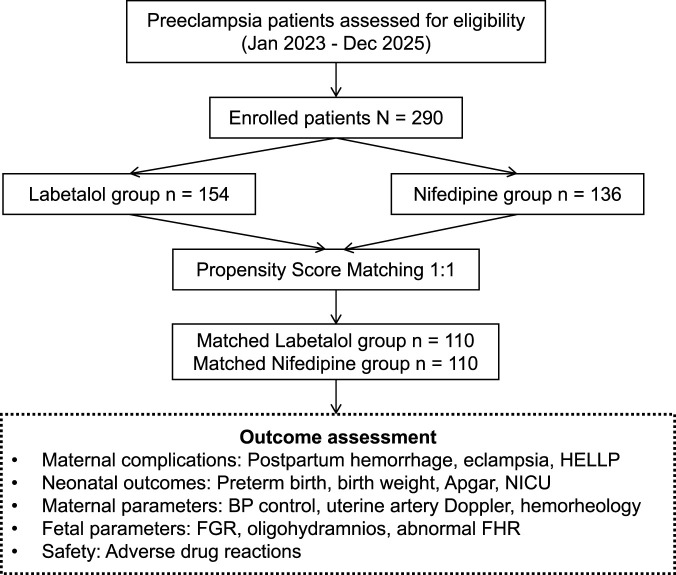
Research process.

This study employed a retrospective cohort design, consecutively enrolling patients with preeclampsia who were admitted for delivery to the Obstetrics Department of our hospital between January 2023 and December 2025. All patients were clinically diagnosed with preeclampsia and received either oral labetalol or oral sustained-release nifedipine as their primary antihypertensive therapy based on the clinicians’ treatment decisions. The choice between labetalol and nifedipine was made at the attending physician’s discretion, influenced by factors such as maternal heart rate, contraindications (e.g., asthma for labetalol, bradycardia for nifedipine), and anticipated tolerability. Clinical data, laboratory results, ultrasound monitoring findings, and maternal-fetal outcomes were collected through review of the electronic medical record system. The specific study process is illustrated in Figure 1.

### 2.2 Inclusion criteria

(1) Gestational age ≥20 weeks; (2) Met the diagnostic criteria of the American College of Obstetricians and Gynecologists (ACOG) (Gestational Hypertension and Preeclampsia, 2020); (3) Had complete clinical data, including blood pressure monitoring, laboratory tests, and delivery outcome records; (4) Received oral antihypertensive therapy for the first time upon admission, with the treatment regimen including either labetalol or sustained-release nifedipine as the primary antihypertensive agent.

### 2.3 Exclusion criteria

(1) Chronic hypertension complicating pregnancy or hypertension with superimposed preeclampsia; (2) Pregnancy complicated by severe cardiac disease; (3) Contraindications or history of allergy to labetalol or nifedipine; (4) Concomitant severe liver or renal dysfunction; (5) Concomitant gestational diabetes mellitus with poor glycemic control; (6) Concomitant active autoimmune diseases; (7) Concomitant severe hematological disorders or coagulation dysfunction; (8) Fetal major structural abnormalities or chromosomal abnormalities; (9) Required switching of antihypertensive medication regimen due to clinical condition during treatment; (10) Incomplete clinical data or loss to follow-up.

### 2.4 Ethical statement

This study was approved by Henan Provincial People’s Hospital’s Ethics Committee and adhered to the Declaration of Helsinki. As a retrospective observational study using anonymized data, informed consent from the pregnant women was waived.

### 2.5 Sample matching

During the screening period, a total of 290 eligible patients with preeclampsia were enrolled in this study, comprising 154 cases in the Labetalol Group and 136 cases in the nifedipine Group. To mitigate selection bias and confounding factors inherent in retrospective studies, propensity score matching (PSM) was employed to match patients from the two groups at a 1:1 ratio. Matching variables included all baseline characteristics presented in Table 1: age (categorized as <35 years or ≥35 years), pre-pregnancy body mass index (normal vs. overweight/obesity), gestational age at onset (≥34 weeks vs. <34 weeks), baseline systolic and diastolic blood pressure, severity of preeclampsia (mild vs. severe), prior obstetric history (history of preeclampsia), education level (as a proxy for socioeconomic status), residence (urban vs. rural), gravidity, parity, multiple pregnancy, number of antenatal visits and regularity of antenatal care, pre-gestational diabetes, and other comorbidities (kidney disease, thyroid disease). A caliper width of 0.02 was set. Following PSM, 110 patients were successfully matched in each group, resulting in a total of 220 patients included in the final statistical analysis. After matching, no statistically significant differences remained between the two groups for any of the matched variables (all P > 0.05), confirming adequate balance.

**TABLE 1:** Baseline characteristics (after PSM).

Variables	Total (n = 220)	Labetalol group (n = 110)	Nifedipine group (n = 110)	Statistics	P	SMD
Age (years), Mean ± SD	31.68 ± 4.85	31.82 ± 4.83	31.55 ± 4.89	t = 0.416	0.678	0.056
Age <35 years, n (%)	162 (73.64)	80 (72.73)	82 (74.55)	χ^2^ = 0.094	0.760	0.041
Age ≥35 years, n (%)	58 (26.36)	30 (27.27)	28 (25.45)			
Pre-pregnancy BMI (kg/m^2^), Mean ± SD	24.25 ± 3.90	24.22 ± 3.77	24.29 ± 4.04	t = −0.140	0.889	0.019
Normal pre-pregnancy weight, n (%)	110 (50.00)	54 (49.09)	56 (50.91)	χ^2^ = 0.073	0.787	0.036
Pre-pregnancy overweight or obesity, n (%)	110 (50.00)	56 (50.91)	54 (49.09)			
Education level, n (%)						
Junior high school or below	49 (22.27)	24 (21.82)	25 (22.73)	Z = −0.118	0.906	0.056
High school/Technical secondary school	85 (38.64)	44 (40.00)	41 (37.27)			
College degree or above	86 (39.09)	42 (38.18)	44 (40.00)			
Residence						
Urban	143 (65.00)	70 (63.64)	73 (66.36)	χ^2^ = 0.180	0.672	0.057
Rural	77 (35.00)	40 (36.36)	37 (33.64)			
Gestational age at onset (weeks), Mean ± SD	33.94 ± 3.13	33.91 ± 3.12	33.96 ± 3.16	t = −0.129	0.898	0.017
Gestational age at onset ≥34 weeks, n (%)	141 (64.09)	71 (64.55)	70 (63.64)	χ^2^ = 0.020	0.888	0.019
Gestational age at onset <34 weeks, n (%)	79 (35.91)	39 (35.45)	40 (36.36)			
Gravidity (times), M (Q1, Q3)	1.00 (1.00, 2.00)	1.00 (1.00, 2.00)	1.00 (1.00, 2.00)	Z = −0.385	0.700	0.056
Parity (times), M (Q1, Q3)	0.00 (0.00, 1.00)	0.00 (0.00, 1.00)	0.00 (0.00, 1.00)	Z = 0.129	0.897	0.019
Primipara	125 (56.82)	62 (56.36)	63 (57.27)	χ^2^ = 0.019	0.892	0.018
Multiple pregnancy, n (%)	18 (8.18)	9 (8.18)	9 (8.18)	χ^2^ = 0.000	1.000	0.000
Number of antenatal visits, M (Q1, Q3)	6.00 (5.00, 8.00)	6.00 (5.00, 8.00)	6.00 (5.00, 8.00)	Z = 0.251	0.802	0.030
Regular antenatal visits, n (%)	178 (80.91)	90 (81.82)	88 (80.00)	χ^2^ = 0.118	0.732	0.046
Severity of preeclampsia						
Mild	88 (40.00)	42 (38.18)	46 (41.82)	χ^2^ = 0.303	0.582	0.074
Severe	132 (60.00)	68 (61.82)	64 (58.18)			​
SBP(mmHg), Mean ± SD	154.85 ± 10.38	154.25 ± 9.67	155.45 ± 11.05	t = −0.857	0.392	0.096
DBP (mmHg), Mean ± SD	98.95 ± 8.79	98.62 ± 8.44	99.27 ± 9.15	t = −0.551	0.582	0.075
Pre-gestational diabetes, n (%)	28 (12.73)	15 (13.64)	13 (11.82)	χ^2^ = 0.164	0.686	0.055
History of kidney disease, n (%)	8 (3.64)	4 (3.64)	4 (3.64)	χ^2^ = 0.000	1.000	0.000
History of thyroid disease, n (%)	15 (6.82)	8 (7.27)	7 (6.36)	χ^2^ = 0.072	0.789	0.036
History of previous preeclampsia, n (%)	10 (4.55)	5 (4.55)	5 (4.55)	χ^2^ = 0.000	1.000	0.000

### 2.6 Treatment regimens

Patients in both groups initiated oral antihypertensive therapy according to routine clinical practice following diagnosis (Gestational Hypertension and Preeclampsia, 2020).

Labetalol Group: The initial dose was oral labetalol hydrochloride 100–200 mg, administered 2–3 times daily. Dosage adjustments were made by the attending physician based on blood pressure control, with a maximum daily dose not exceeding 2400 mg.

Nifedipine Group: The initial dose was oral sustained-release nifedipine tablets 10–20 mg, administered every 12 h. Dosage was adjusted based on blood pressure control, with a maximum daily dose not exceeding 120 mg.

Both groups received foundational treatment based on disease severity and guideline recommendations: (1) Intravenous magnesium sulfate for seizure prophylaxis when clinically indicated; (2) Adjunctive use of aspirin or low-molecular-weight heparin based on assessment of platelet counts and coagulation function. However, the use of these adjunctive therapies was not protocol-driven or standardized across all patients. The decision to administer magnesium sulfate, aspirin, or low-molecular-weight heparin was made by the attending physician based on individual clinical judgment and evolving patient conditions, which may introduce treatment heterogeneity and residual confounding despite propensity score matching.

### 2.7 Clinical outcome measures

#### 2.7.1 Primary outcome measures

1. Maternal complications: Including the incidence of postpartum hemorrhage, eclampsia, HELLP syndrome, placental abruption, and acute kidney injury. For the logistic regression analysis, a composite adverse maternal-fetal outcome was defined as any of the following: postpartum hemorrhage, preterm birth (<37 weeks), eclampsia, placental abruption, HELLP syndrome, or perinatal death.
2. Neonatal outcomes: Preterm birth rate (<37 weeks), early preterm birth rate (<34 weeks), mean birth weight, proportion of very low birth weight infants (<1,500 g), Apgar scores (1-min, 5-min), incidence of neonatal asphyxia, NICU admission rate, fetal distress, intraventricular hemorrhage, necrotizing enterocolitis, perinatal mortality rate, *etc.*,

#### 2.7.2 Secondary outcome measures

Maternal parameters: (1) Blood pressure control: Systolic blood pressure (SBP), diastolic blood pressure (DBP), rate of achieving target blood pressure (<135/85 mmHg). (2) Uterine artery blood flow: Systolic/Diastolic (S/D) ratio, Pulsatility Index (PI), Resistance Index (RI). (3) Hemodynamics: Plasma viscosity, high-shear whole blood viscosity, hematocrit. (4) Safety indicators: Incidence of adverse drug reactions (e.g., headache, facial flushing, tachycardia, nausea, dizziness).

Fetal and neonatal parameters: Incidence of fetal growth restriction (FGR), incidence of oligohydramnios, and rate of abnormal fetal heart rate monitoring.

### 2.8 Statistical analysis

Statistical analyses were performed using SPSS 26.0. Continuous variables are presented as mean ± standard deviation (SD) and were compared using the t-test or Mann-Whitney U test, while categorical variables are presented as frequencies (%) and were compared using the χ^2^ test or Fisher’s exact test. To reduce confounding bias, 1:1 nearest-neighbor PSM was applied with a caliper width of 0.02. Post-matching balance was assessed using standardized mean differences (SMD), with an SMD < 0.1 indicating acceptable balance. The pre-specified primary outcomes of this study were maternal complications and neonatal outcomes, and the main conclusions are based on the analysis of primary outcomes. Secondary outcomes (including uterine artery blood flow parameters, hemodynamic indices, and adverse drug reactions) were considered exploratory. No adjustment for multiple comparisons was applied to these secondary outcomes to avoid increasing type II error due to controlling for type I error, and the results should be interpreted with caution. Variables with P < 0.05 in univariate analysis were entered into a multivariate logistic regression model to identify independent predictors of adverse maternal-fetal outcomes. A two-tailed P < 0.05 was considered statistically significant unless otherwise specified.

## 3 Results

### 3.1 Baseline characteristics after PSM matching

Table 1 presents a comparison of the baseline clinical characteristics between the Labetalol Group and the nifedipine Group following Propensity Score Matching. The results showed no statistically significant differences between the two groups in terms of age, pre-pregnancy BMI, education level, place of residence, gestational age at onset, gravidity, parity, multiple pregnancy, prenatal care attendance, severity of preeclampsia, baseline blood pressure values, and comorbid conditions (P > 0.05).

### 3.2 Blood pressure control and hemodynamic parameters

Table 2 showed that after treatment, systolic blood pressure, DBP, uterine artery S/D ratio, PI, RI, as well as plasma viscosity, whole blood viscosity, and hematocrit were all significantly improved compared to pretreatment values in both groups (P < 0.05). However, there were no statistically significant differences between the two groups (P > 0.05), suggesting that both antihypertensive regimens have comparable efficacy in controlling blood pressure and improving hemodynamic parameters.

**TABLE 2:** Blood pressure control and hemodynamic parameters, Mean ± SD.

Time	Variables	Total (n = 220)	Labetalol group (n = 110)	Nifedipine group (n = 110)	Statistics	P
Pre-treatment	SBP (mmHg)	154.85 ± 10.38	154.25 ± 9.67	155.45 ± 11.05	t = −0.857	0.392
Post-treatment	SBP (mmHg)	129.95 ± 9.58*	129.83 ± 9.76*	130.08 ± 9.45*	t = −0.197	0.844
Pre-treatment	DBP (mmHg)	98.95 ± 8.79	98.62 ± 8.44	99.27 ± 9.15	t = −0.551	0.582
Post-treatment	DBP (mmHg)	79.53 ± 9.10*	79.58 ± 9.06*	79.47 ± 9.19*	t = 0.089	0.929
Pre-treatment	S/D ratio	2.88 ± 0.45	2.85 ± 0.42	2.91 ± 0.48	t = −0.883	0.378
Post-treatment	S/D ratio	2.40 ± 0.36*	2.38 ± 0.35*	2.42 ± 0.37*	t = −0.620	0.536
Pre-treatment	PI	1.13 ± 0.20	1.12 ± 0.18	1.15 ± 0.21	t = −1.019	0.309
Post-treatment	PI	0.90 ± 0.15*	0.89 ± 0.15*	0.91 ± 0.16*	t = −1.294	0.197
Pre-treatment	RI	0.88 ± 0.18	0.88 ± 0.17	0.89 ± 0.19	t = −0.004	0.997
Post-treatment	RI	0.70 ± 0.16*	0.70 ± 0.17*	0.69 ± 0.15*	t = 0.900	0.369
Pre-treatment	Plasma viscosity (mPa·s)	1.86 ± 0.24	1.85 ± 0.22	1.88 ± 0.25	t = −0.620	0.536
Post-treatment	Plasma viscosity (mPa·s)	1.54 ± 0.20*	1.52 ± 0.18*	1.55 ± 0.21*	t = −1.136	0.257
Pre-treatment	High-shear whole blood viscosity (mPa·s)	4.65 ± 0.61	4.62 ± 0.57	4.68 ± 0.64	t = −0.733	0.464
Post-treatment	High-shear whole blood viscosity (mPa·s)	3.77 ± 0.49*	3.79 ± 0.48*	3.75 ± 0.52*	t = 0.557	0.578
Pre-treatment	Hematocrit (%)	38.70 ± 4.53	38.57 ± 4.26	38.82 ± 4.81	t = −0.409	0.683
Post-treatment	Hematocrit (%)	35.69 ± 3.99*	35.59 ± 3.81*	35.80 ± 4.17*	t = −0.370	0.711

*P < 0.05 compared with pre-treatment value in the same group.

### 3.3 Maternal complications

Table 3 showed that the rate of postpartum hemorrhage was significantly lower in the Labetalol Group compared to the nifedipine Group (8.8% vs. 17.0%, P = 0.043). There were no statistically significant differences between the two groups in the incidence of other complications, such as eclampsia, placental abruption, HELLP syndrome, acute kidney injury, pulmonary edema, or retinopathy (P > 0.05).

**TABLE 3:** Maternal complications, n (%).

Variables	Total (n = 220)	Labetalol group (n = 110)	Nifedipine group (n = 110)	Statistics	P	OR (95% CI)
*Postartum* hemorrhage	28 (12.73)	9 (8.18)	19 (17.27)	χ^2^ = 4.092	0.043	0.427 (0.184–0.991)
Eclampsia	5 (2.27)	2 (1.82)	3 (2.73)	χ^2^ = 0.000	1.000	​
Placental abruption	12 (5.45)	5 (4.55)	7 (6.36)	χ^2^ = 0.353	0.553	​
HELLP syndrome	10 (4.55)	4 (3.64)	6 (5.45)	χ^2^ = 0.419	0.517	​
Acute kidney injury	7 (3.18)	3 (2.73)	4 (3.64)	χ^2^ = 0.000	1.000	​
Pulmonary edema	2 (0.91)	1 (0.91)	1 (0.91)	χ^2^ = 0.000	1.000	​
Retinopathy	14 (6.36)	6 (5.45)	8 (7.27)	χ^2^ = 0.305	0.581	​

### 3.4 Fetal and neonatal outcomes

Table 4 showed that the Labetalol Group had a significantly lower rate of preterm birth (<37 weeks) compared to the nifedipine Group (24.6% vs. 37.3%, P = 0.041). Neonatal birth weight was significantly higher in the Labetalol Group (2933.95 ± 803.23 g vs. 2541.35 ± 631.41 g, P < 0.001). However, this observed difference in birth weight may be partially confounded by the difference in preterm birth rates and residual disease severity, despite propensity score matching. No statistically significant differences were observed between the groups in other parameters, including fetal growth restriction, oligohydramnios, abnormal fetal heart rate monitoring, early preterm birth (<34 weeks), very low birth weight, Apgar scores, fetal distress, NICU admission rate, neonatal complications, and perinatal mortality (P > 0.05).

**TABLE 4:** Fetal and neonatal outcomes.

Variables	Total (n = 220)	Labetalol group (n = 110)	Nifedipine group (n = 110)	Statistics	P	OR (95% CI)/Mean difference (95% CI)
Fetal growth restriction, n (%)	27 (12.27)	12 (10.91)	15 (13.64)	χ^2^ = 0.380	0.538	​
Oligohydramnios, n (%)	20 (9.09)	9 (8.18)	11 (10.00)	χ^2^ = 0.220	0.639	​
Abnormal fetal heart rate monitoring, n (%)	40 (18.18)	18 (16.36)	22 (20.00)	χ^2^ = 0.489	0.484	​
Preterm birth (<37 weeks), n (%)	68 (30.91)	27 (24.55)	41 (37.27)	χ^2^ = 4.172	0.041	0.547 (0.306–0.979)
Early preterm birth (<34 weeks), n (%)	20 (9.09)	8 (7.27)	12 (10.91)	χ^2^ = 0.880	0.348	​
Very low birth weight infant (<1500 g), n (%)	10 (4.55)	4 (3.64)	6 (5.45)	χ^2^ = 0.419	0.517	​
Neonatal birth weight, Mean ± SD	2737.65 ± 747.16	2933.95 ± 803.23	2541.35 ± 631.41	t = 4.030	<0.001	392.59 (200.60–584.59)
1-min Apgar score (points), Mean ± SD	8.15 ± 1.17	8.25 ± 1.36	8.05 ± 0.93	t = 1.275	0.204	​
5-min Apgar score (points), Mean ± SD	9.10 ± 0.54	9.05 ± 0.54	9.15 ± 0.54	t = −1.251	0.212	​
Neonatal asphyxia, n (%)	15 (6.82)	6 (5.45)	9 (8.18)	χ^2^ = 0.644	0.422	​
NICU admission, n (%)	50 (22.73)	22 (20.00)	28 (25.45)	χ^2^ = 0.932	0.334	​
Fetal distress, n (%)	19 (8.64)	8 (7.27)	11 (10.00)	χ^2^ = 0.518	0.471	​
Intraventricular hemorrhage, n (%)	5 (2.27)	2 (1.82)	3 (2.73)	χ^2^ = 0.000	1.000	​
Necrotizing enterocolitis, n (%)	3 (1.36)	1 (0.91)	2 (1.82)	χ^2^ = 0.000	1.000	​
Perinatal death, n (%)	5 (2.27)	2 (1.82)	3 (2.73)	χ^2^ = 0.000	1.000	​

### 3.5 Adverse drug reactions

Table 5 showed that the nifedipine Group had significantly higher rates of headache (16.36% vs. 7.27%, P = 0.037) and facial flushing (13.64% vs. 4.55%, P = 0.019) compared to the Labetalol Group. No statistically significant differences were observed between the two groups in the incidence of other adverse reactions such as tachycardia, nausea, dizziness, or hypotension (P > 0.05).

**TABLE 5:** Adverse drug reactions, n (%).

Variables	Total (n = 220)	Labetalol group (n = 110)	Nifedipine group (n = 110)	Statistics	P	OR (95% CI)
Headache	26 (11.82)	8 (7.27)	18 (16.36)	χ2 = 4.362	0.037	0.401 (0.166–0.966)
Facial flushing	20 (9.09)	5 (4.55)	15 (13.64)	χ2 = 5.500	0.019	0.302 (0.106–0.861)
Tachycardia	15 (6.82)	6 (5.45)	9 (8.18)	χ2 = 0.644	0.422	​
Nausea	10 (4.55)	4 (3.64)	6 (5.45)	χ2 = 0.419	0.517	​
Dizziness	15 (6.82)	7 (6.36)	8 (7.27)	χ2 = 0.072	0.789	​
Hypotension	7 (3.18)	3 (2.73)	4 (3.64)	χ2 = 0.000	1.000	​
Any adverse reaction	60 (27.27)	25 (22.73)	35 (31.82)	χ2 = 2.292	0.130	​

### 3.6 Adverse maternal-fetal outcomes

A composite adverse maternal-fetal outcome was defined as the occurrence of any of the following: postpartum hemorrhage, preterm birth (<37 weeks), eclampsia, placental abruption, HELLP syndrome, or perinatal death. Based on this composite endpoint, the 220 patients were divided into an adverse outcome group (n = 78) and a no adverse outcome group (n = 142). Table 6 compares the baseline characteristics between the two groups. Univariate analysis revealed that the proportions of pre-gestational diabetes mellitus, primiparity, multiple pregnancy, pre-pregnancy overweight or obesity, severe preeclampsia, onset of preeclampsia at <34 weeks gestation, and maternal age ≥35 years were significantly higher in the adverse outcome group compared to the no adverse outcome group (P < 0.05). No statistically significant difference was observed in the distribution of antihypertensive medications between the two groups (P > 0.05).

**TABLE 6:** Baseline characteristics between the adverse maternal-fetal outcome group and the no adverse outcome group.

Variables	Total (n = 220)	No adverse outcome group (n = 142)	Adverse outcome group (n = 78)	Statistics	P	OR (95% CI)
Age, n (%)						
<35 years	162 (73.64)	112 (78.87)	50 (64.10)	χ^2^ = 5.658	0.017	2.091 (1.1352–3.861)
≥35 years	58 (26.36)	30 (21.13)	28 (35.90)			
Pre-pregnancy BMI						
Normal pre-pregnancy weight, n (%)	110 (50.00)	80 (56.34)	30 (38.46)	χ^2^ = 6.436	0.011	2.065 (1.175–3.629)
Pre-pregnancy overweight or obesity, n (%)	110 (50.00)	62 (43.66)	48 (61.54)			
Education level, n (%)						
Junior high school or below	49 (22.27)	33 (23.24)	16 (20.51)	Z = −0.727	0.467	​
High school/Technical secondary school	85 (38.64)	56 (39.44)	29 (37.18)			​
College degree or above	86 (39.09)	53 (37.32)	33 (42.31)			​
Residence						
Urban	143 (65.00)	93 (65.49)	50 (64.10)	χ^2^ = 0.043	0.836	​
Rural	77 (35.00)	49 (34.51)	28 (35.90)			​
Gestational age at onset, n (%)						
≥34 weeks	141 (64.09)	106 (74.65)	35 (44.87)	χ^2^ = 19.395	<0.001	3.617 (2.016–6.492)
<34 weeks	79 (35.91)	36 (25.35)	43 (55.13)			
Primipara	125 (56.82)	73 (51.41)	52 (66.67)	χ^2^ = 4.777	0.029	0.529 (0.298–0.939)
Multiple pregnancy, n (%)	18 (8.18)	6 (4.23)	12 (15.38)	χ^2^ = 8.346	0.004	0.243 (0.087–0.675)
Number of antenatal visits, M (Q1, Q3)	6.00 (5.00, 8.00)	6.00 (5.00, 8.00)	7.00 (5.00, 8.00)	Z = −1.296	0.195	​
Regular antenatal visits, n (%)	178 (80.91)	116 (81.69)	62 (79.49)	χ^2^ = 0.158	0.691	​
Severity of preeclampsia						
Mild	88 (40.00)	71 (50.00)	17 (21.79)	χ^2^ = 16.688	<0.001	3.588 (1.911–6.739)
Severe	132 (60.00)	71 (50.00)	61 (78.21)			
Pre-gestational diabetes, n (%)	28 (12.73)	12 (8.45)	16 (20.51)	χ^2^ = 6.595	0.010	0.358 (0.160–0.802)
History of kidney disease, n (%)	8 (3.64)	5 (3.52)	3 (3.85)	χ^2^ = 0.000	1.000	​
History of thyroid disease, n (%)	15 (6.82)	9 (6.34)	6 (7.69)	χ^2^ = 0.145	0.703	​
History of previous preeclampsia, n (%)	10 (4.55)	6 (4.23)	4 (5.13)	χ^2^ = 0.000	1.000	​
Antihypertensive medication, n (%)						
Labetalol	110 (50.00)	75 (52.82)	35 (44.87)	χ^2^ = 1.271	0.260	​
Nifedipine	110 (50.00)	67 (47.18)	43 (55.13)			​

### 3.7 Multivariate logistic regression analysis

Variables that were statistically significant in the univariate analysis from Table 6 (pre-gestational diabetes mellitus, primiparity, multiple pregnancy, pre-pregnancy overweight or obesity, severe preeclampsia, onset of preeclampsia at <34 weeks gestation, and maternal age ≥35 years) were entered into a multivariate logistic regression model. The results, presented in Table 7, showed that maternal age ≥35 years (OR = 2.091, 95% CI: 1.131–3.872, P = 0.018), pre-pregnancy overweight or obesity (OR = 2.064, 95% CI: 1.180–3.655, P = 0.012), onset of preeclampsia at <34 weeks gestation (OR = 3.618, 95% CI: 2.027–6.545, P < 0.001), primiparity (OR = 1.890, 95% CI: 1.071–3.390, P = 0.030), multiple pregnancy (OR = 4.121, 95% CI: 1.530–12.288, P = 0.007), severe preeclampsia (OR = 3.588, 95% CI: 1.944–6.894, P < 0.001), and pre-gestational diabetes mellitus (OR = 2.796, 95% CI: 1.254–6.391, P = 0.013) were all independent risk factors for adverse maternal-fetal outcomes. The model demonstrated good fit (Nagelkerke R^2^ = 0.305). Furthermore, the type of antihypertensive medication did not enter the final model in the regression analysis, suggesting that it was not an independent predictor of adverse maternal-fetal outcomes in this study population.

**TABLE 7:** Multivariate logistic regression analysis.

Variable	Estimate	S.E	OR (95%CI)	Z	P
Maternal age ≥35 years	0.738	0.313	2.091 (1.131–3.872)	2.356	0.018
Pre-pregnancy overweight or obesity	0.725	0.288	2.064 (1.180–3.655)	2.519	0.012
Gestational age at onset <34 weeks	1.286	0.298	3.618 (2.027–6.545)	4.309	<0.001
Primipara	0.637	0.293	1.890 (1.071–3.390)	2.173	0.030
Multiple pregnancy	1.416	0.522	4.121 (1.530–12.288)	2.713	0.007
Severe preeclampsia	1.278	0.322	3.588 (1.944–6.894)	3.974	<0.001
Pre-gestational diabetes	1.028	0.412	2.796 (1.254–6.391)	2.496	0.013

## 4 Discussion

Preeclampsia, as a pregnancy-specific multisystem disorder, presents a clinical challenge where the choice of antihypertensive strategy directly impacts maternal and fetal prognosis. This study systematically compared the effects of two first-line oral antihypertensive regimens—labetalol and sustained-release nifedipine—on maternal-fetal outcomes in patients with preeclampsia. We found that while both agents demonstrated comparable efficacy in blood pressure control and hemodynamic improvement, significant differences emerged regarding postpartum hemorrhage, preterm birth, and adverse drug reactions. This finding holds substantial practical significance for guiding individualized clinical medication. Regarding blood pressure control, both the Labetalol Group and the nifedipine Group in our study showed significant reductions in systolic and DBP post-treatment compared to baseline, with no statistically significant difference between the groups. This suggests that both medications effectively manage hypertension in preeclamptic patients. A randomized controlled trial by Webster et al. (2017) (n = 112) directly comparing labetalol and nifedipine for chronic hypertension in pregnancy reported no significant difference in blood pressure control, with mean achieved blood pressures of 134/84 mmHg versus 134/85 mmHg, respectively (mean difference: systolic 0.3 mmHg, 95% CI -2.8 to 3.4; diastolic −1.9 mmHg, 95% CI -4.1 to 0.3). This conclusion aligns with the findings of Leonard et al. (2025), whose analysis of a large cohort of 6,724 pregnant women (5,504 initiated labetalol, 1,220 initiated nifedipine) in the United States clearly indicated that labetalol and nifedipine possess similar efficacy and safety profiles in the treatment of chronic hypertension during pregnancy: the primary effectiveness outcome (severe preeclampsia/eclampsia, medically indicated preterm birth, placental abruption, or stillbirth) occurred in 42% of the labetalol group versus 44% of the nifedipine group (adjusted RR 1.03, 95% CI: 0.96–1.11), and the safety outcome (small for gestational age) occurred in 13% versus 12%, respectively (adjusted RR 0.98, 95% CI: 0.82–1.16). The equivalence in controlling systolic and DBP observed in our study corroborates the results of these larger trials, further supporting the rationale behind current international guidelines that list both agents as first-line options.

Uterine artery hemodynamic parameters are critical indicators reflecting placental perfusion status and are closely associated with the severity of preeclampsia and fetal prognosis (Pedroso et al., 2018; Song et al., 2019). After treatment, both groups showed significant improvements in uterine artery S/D ratio, PI, and RI from baseline, with no significant differences between them. Research by Hassan et al. (Has et al., 2021), utilizing Doppler ultrasound evaluation, found that labetalol effectively maintains uteroplacental perfusion while lowering blood pressure, with its effect on improving uterine artery PI and RI being comparable to that of nifedipine. This finding aligns with our observation that uterine artery blood flow parameters significantly improved in both groups without inter-group differences. The increased resistance in uterine arteries among preeclamptic patients is largely attributable to systemic maternal vasospasm/vasoconstriction, leading to abnormal perfusion pressure (Ridder et al., 2019; Qu and Khalil, 2020). Once both medications successfully lower maternal blood pressure to the target range, the elevated uterine artery resistance state directly caused by hypertension is consequently alleviated. Labetalol, an α/β-adrenergic receptor blocker, induces direct vasodilation by blocking α1 receptors while simultaneously slowing heart rate and reducing myocardial oxygen demand through β-blockade (Woolston et al., 2022). Nifedipine, a dihydropyridine calcium channel blocker, directly inhibits calcium influx into vascular smooth muscle cells, potently dilating peripheral arterioles, and clinical studies have not shown negative effects on uteroplacental blood flow (Everett and Lees, 2012). Although labetalol and nifedipine have different mechanisms of action, they both effectively reduce systemic vascular resistance in preeclamptic patients through the common pathway of lowering blood pressure and inducing vasodilation. When they achieve similar levels of blood pressure control, they demonstrate clinical equivalence in improving uterine artery Doppler parameters.

*Postartum* hemorrhage is a major complication during the delivery period in patients with preeclampsia and poses a serious threat to maternal safety. This study found that the rate of postpartum hemorrhage in the Labetalol Group was significantly lower than that in the nifedipine Group, a finding of considerable clinical importance. Research by Ajit Kumar Gupta (Ajit Kumar Gupta, 2026) indicated that in managing gestational hypertension, oral labetalol was associated with significantly reduced blood loss during delivery and a lower risk of postpartum hemorrhage, potentially related to labetalol’s relatively milder vasodilatory effect which may help maintain better uterine tone. The calcium channel blocking action of nifedipine could inhibit uterine smooth muscle contraction, whereas labetalol’s alpha-receptor blocking effect, while dilating vessels, has a relatively smaller impact on uterine contractility (Sondgeroth et al., 2025). Furthermore, the potent systemic vasodilation caused by nifedipine might lead to sustained dilation of the uterine vascular bed postpartum, affecting uterine contraction and the hemostatic process (Zamani et al., 2024). This finding suggests that for preeclamptic patients with high-risk factors for postpartum hemorrhage, labetalol may represent a safer choice. Preterm birth is a critical factor requiring careful consideration in the management of preeclampsia, where one goal of antihypertensive therapy is to prolong gestation as much as possible while controlling the maternal condition. The significantly lower rate of preterm birth observed in the Labetalol Group compared to the nifedipine Group in this study supports the advantage of labetalol in extending gestational age. A large-scale systematic review and network meta-analysis provides high-level evidence for this, incorporating 23 trials with a total of 3,989 patients and showing that labetalol reduced the risk of preterm birth by 32% compared to nifedipine (Hup et al., 2025). This result aligns closely with our finding of a significantly lower preterm birth rate in the Labetalol Group. The same meta-analysis also indicated that labetalol has advantages in reducing the risk of developing preeclampsia. These findings suggest that labetalol, potentially through more stable hemodynamic control, might mitigate the impact of blood pressure fluctuations on the uteroplacental unit, thereby delaying disease progression and allowing more time for fetal maturation. In contrast, although nifedipine is equally effective at lowering blood pressure, its faster onset and potent vasodilatory properties could lead to greater blood pressure variability or reflex activation of the sympathetic nervous system, causing tachycardia—factors that might be less conducive to prolonging pregnancy (van Winden et al., 2022).

Drug safety is a key consideration in selecting antihypertensive regimens. This study found that headache and facial flushing occurred significantly more often in the nifedipine Group than the Labetalol Group, likely due to nifedipine’s vasodilatory effects. A meta-analysis by George et al. (2022) comprising 22 randomized controlled trials with 2,595 participants indicated that nifedipine was significantly superior to other antihypertensive agents (labetalol, hydralazine, methyldopa) in lowering blood pressure, with both the mean time to achieve target blood pressure and the number of doses required being significantly lower in the nifedipine group (P < 0.05). Furthermore, a randomized controlled trial by Easterling et al. (2019) involving 894 pregnant women with severe hypertension compared the efficacy of oral nifedipine, labetalol, and methyldopa. The primary outcome of achieving blood pressure control (120–150/70–100 mmHg) within 6 h without adverse outcomes occurred in 84% (249/298) of the nifedipine group, 77% (228/295) of the labetalol group, and 76% (230/301) of the methyldopa group. The primary outcome was significantly more common in the nifedipine group than in the methyldopa group (P = 0.03), but the difference between nifedipine and labetalol did not reach statistical significance (84% vs. 77%, P = 0.05). Zhu et al. (2021) compared calcium channel blockers and beta-blockers in the treatment of hypertension, noting that the vasodilatory effects of calcium channel blockers can lead to headache, facial flushing, palpitations, peripheral edema, and hypotension; such vasodilatory side effects are more common with dihydropyridine calcium channel blockers. A prospective study by Solanki et al. (2021) also explicitly identified headache, facial flushing, palpitations, peripheral edema, and hypotension as the main side effects of calcium channel blockers. The significantly higher rates of headache and facial flushing observed in the nifedipine Group in our study are consistent with the conclusions of these systematic reviews. Although these adverse reactions are often mild, they may affect patient treatment adherence, thereby potentially impacting blood pressure control. Labetalol, due to its combined alpha and beta-blocking action, counteracts the reflex tachycardia caused by alpha-blockade alone, resulting in more stable hemodynamic changes—a pharmacological property that explains its better tolerability (Chaemsaithong et al., 2024).

The Labetalol Group had a significantly lower preterm birth rate and higher neonatal birth weight than the nifedipine Group. No significant differences were found between groups for other indicators, including fetal growth restriction, oligohydramnios, abnormal fetal heart rate monitoring, Apgar scores, fetal distress, or NICU admission rate. Research by Ajit Kumar Gupta (Ajit Kumar Gupta, 2026) similarly reported a higher mean neonatal birth weight in the labetalol group. However, the observed difference in birth weight in our study should be interpreted with caution, as it may be partially attributable to the significantly lower preterm birth rate in the labetalol group (24.6% vs. 37.3%, P = 0.041). Additionally, despite propensity score matching, residual confounding from unmeasured factors such as disease severity at enrollment or differences in gestational age at delivery cannot be fully excluded. Therefore, while labetalol appears associated with more favorable neonatal growth parameters, causality cannot be firmly established from this retrospective analysis.

Multivariate logistic regression analysis further identified independent risk factors for adverse maternal-fetal outcomes, including maternal age ≥35 years, pre-pregnancy overweight or obesity, onset of preeclampsia at <34 weeks gestation, primiparity, multiple pregnancy, severe preeclampsia, and pre-gestational diabetes mellitus. Recognition of these factors aids clinicians in the early identification and targeted management of high-risk patients (Ozer et al., 2025; Li et al., 2025). Onset at <34 weeks as a predictive factor aligns with the conclusions of a large cohort study by Venkatesh et al. (2020), which confirmed the prognostic value of this gestational age threshold for adverse maternal and neonatal outcomes. The high-risk nature of severe preeclampsia and multiple pregnancy is well established. Jikamo et al. (2022) found that severe features increased the risk of adverse perinatal outcomes by 46%, while a large cohort study confirmed significantly higher risks for most adverse outcomes in twin pregnancies complicated by preeclampsia, excluding stillbirth and neonatal asphyxia (Chai et al., 2025). Risk factors such as maternal age ≥35 years and pre-pregnancy overweight or obesity align with the 2020 ACOG Guideline (Gestational Hypertension and Preeclampsia, 2020). Notably, the type of antihypertensive medication did not enter the final regression model, suggesting that after controlling for other confounding factors, labetalol and nifedipine did not show a significant difference in the overall risk of adverse maternal-fetal outcomes. This aligns with the observed equivalence of the two drugs in blood pressure control and hemodynamic improvement. However, labetalol still demonstrated advantages in specific outcomes such as postpartum hemorrhage and preterm birth, which may be related to the more complex mechanisms underlying these outcomes, involving various pathophysiological processes beyond simple blood pressure control. Given the observational nature of this study, these findings should be considered hypothesis-generating rather than definitive. The findings of this study support the preferential selection of labetalol in specific clinical scenarios, particularly when the therapeutic goals include prolonging gestation, reducing preterm birth, or when the patient has high-risk factors for postpartum hemorrhage. Nevertheless, clinical decision-making must remain individualized, taking into account patient comorbidities, drug contraindications, and treatment response. For instance, in patients with concomitant asthma or chronic obstructive pulmonary disease, the beta-blocking effect of labetalol may warrant cautious use; for patients with bradycardia or heart block, nifedipine might be more suitable.

## 5 Study limitations

As a retrospective cohort study, despite using PSM to control for measured confounders, the potential for unmeasured confounding factors remains. Inherent limitations of retrospective designs include selection bias, information bias related to data completeness, and the inability to establish causality. Although PSM improves comparability, it cannot fully replace the rigor of randomization. These factors should be considered when interpreting the findings. Although propensity score matching reduced confounding, the post-matching sample size (n = 220) is modest. Consequently, the study is underpowered to detect differences in rare but clinically important outcomes such as eclampsia, HELLP syndrome, and perinatal mortality. Findings for these outcomes should be interpreted cautiously. Furthermore, the study did not perform a standardized analysis of medication dosages, and different dosages could potentially influence outcomes. Additionally, the absence of long-term follow-up limits evaluation of the drugs’ effects on maternal and fetal prognosis. Additionally, the use of a composite adverse maternal-fetal outcome in logistic regression analysis may obscure differential effects of antihypertensive regimens on individual outcome components. Future large-scale prospective randomized controlled trials are needed to validate these findings and assess the impact of different antihypertensive strategies on long-term cardiovascular and metabolic outcomes in patients with preeclampsia.

## 6 Conclusion

In this retrospective cohort study, both labetalol and nifedipine demonstrated comparable efficacy in blood pressure control and improvement of uteroplacental blood flow. However, labetalol was associated with a lower incidence of postpartum hemorrhage and preterm birth, as well as fewer vasodilatory adverse effects (headache and facial flushing). These findings suggest potential advantages of labetalol over nifedipine in specific maternal and neonatal outcomes, but causal inference is limited by the observational design. Future randomized controlled trials are needed to confirm these associations.

## Data Availability

The original contributions presented in the study are included in the article/supplementary material, further inquiries can be directed to the corresponding author.
